# Re-evaluating the Systematics of *Dendrolycopodium* Using Restriction-Site Associated DNA-Sequencing

**DOI:** 10.3389/fpls.2022.912080

**Published:** 2022-06-09

**Authors:** Alaina R. Petlewski, Duncan A. Hauser, Min Kim, Jeremy Schmutz, Jane Grimwood, Fay-Wei Li

**Affiliations:** ^1^Boyce Thompson Institute, Ithaca, NY, United States; ^2^Plant Biology Section, Cornell University, Ithaca, NY, United States; ^3^HudsonAlpha Institute for Biotechnology, Huntsville, AL, United States; ^4^United States Department of Energy, Joint Genome Institute, Berkeley, CA, United States

**Keywords:** hybridization, Lycopodiaceae, biogeography, *Dendrolycopodium*, *Lycopodium*, RADseq, evolution

## Abstract

The genus *Dendrolycopodium* (Lycopodiaceae) includes four to five species across North America and East Asia. Species identification in *Dendrolycopodium* is difficult due to limited or inconsistent characters. In addition, plants with intermediate morphologies regularly occur, potentially indicative of interspecific hybridization. To determine the species relationships in *Dendrolycopodium* and investigate the existence of hybrids, we generated a draft genome assembly for *D. obscurum* and carried out double-digest restriction-site associated DNA sequencing (RADSeq) on 86 *Dendrolycopodium* specimens. Our sampling includes all the described species and 11 individuals with intermediate morphology. We find that the genus can be divided into four clades that largely correspond to the described taxa, as well as evidence of interspecific hybridization. Within these clades, our STRUCTURE analysis suggests that there are multiple finer subgroups, with evidence of hybridization and introgression between these subgroups. Given the limited availability of specimens collected from Asia, the status of the various Asian species remains uncertain and will require further study. In summary, our study confirms several hybrid relationships in *Dendrolycopodium* and provides a clear phylogenetic framework for future taxonomic revision.

## Introduction

Lycophytes are a clade of vascular plants sister to euphyllophytes (ferns + seed plants). While this group has a rich fossil record, extant lycophytes are constrained to three orders: Selaginellales, Isoëtales, and Lycopodiales (PPG, 2016). Of these, the monogeneric Selaginellales and Isoëtales have garnered the most research attention, with the former being used extensively for studying evo-devo and desiccation tolerance (VanBuren et al., 2018; Spencer et al., 2021) and the latter for its independent origin of CAM photosynthesis and well-documented, rampant hybridization (Szovenyi et al., 2021). The recent publications of *Selaginella* and *Isoëtes* genomes has also brought these two lineages into the modern genomic era (Banks et al., 2011; VanBuren et al., 2018; Xu et al., 2018; Wickell et al., 2021). Lycopodiales, however, are both the most diverse (16 genera across three subfamilies) and least studied order, receiving relatively sparse research and completely lacking genomic resources. Furthermore, Lycopodiales differ significantly from the other groups of lycophytes in several ways. For example, they are homosporous, and two of the three subfamilies produce cryptic, subterranean mycoheterotrophic gametophytes. They are also known for their large genomes, which has complicated the development of genomic resources (Szovenyi et al., 2021). Additionally, they have few reliable morphological characteristics on which to base species distinctions. These factors combined mean that there is an unexplored world of diversity and evolutionary history in the group, which now can begin to be revealed with the use of modern phylogenomic tools.

**FIGURE 1:**
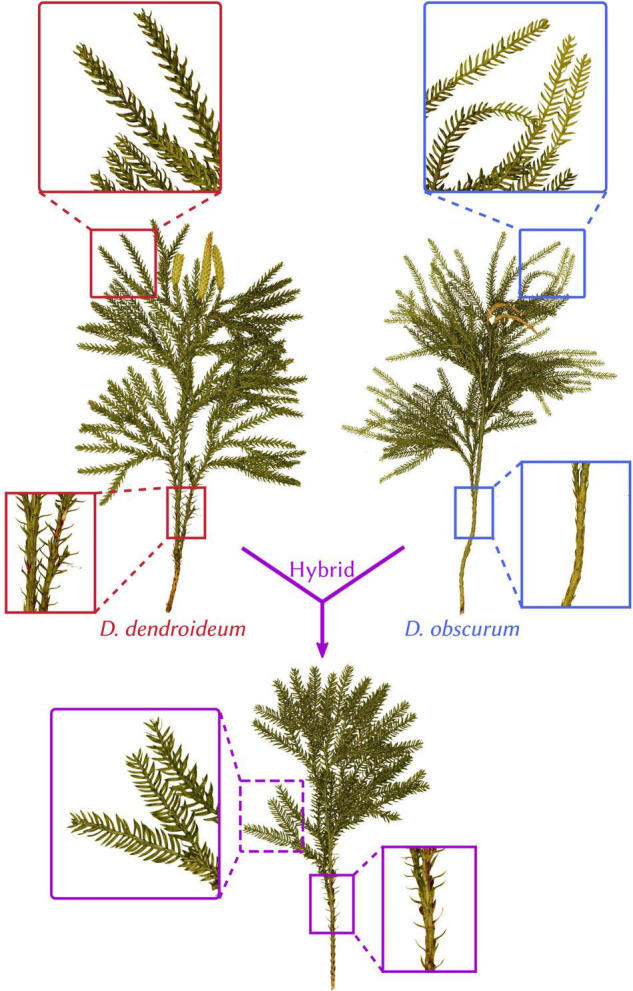
Morphology and hybrid relationship in *Dendrolycopodium*. *Dendrolycopodium dendroideum, D. obscurum*, and their hybrid (Dsp2) exhibit intermingling diagnostic characters (leaf arrangement and divergence angle). All plants were collected from McLean Bogs in New York (42.54795, –76.26633).

Since Linnaeus described the first species (*Dendrolycopodium obscurum*, formerly *Lycopodium obscurum*) in 1753, this genus has been a matter of much debate amongst botanists. Initially considered part of genus *Lycopodium*, in the past 50 years this group has been elevated to generic rank and taxonomists have since recognized five species: *Dendrolycopodium obscurum*, *D. dendroideum, D. hickeyi, D. juniperoideum*, and, most recently, *D. verticale* (Linnaeus, 1753; Michaux, 1803; Eaton, 1890; Hickey, 1977, 1978; Wagner et al., 1989; Haines, 2003; Zhou and Zhang, 2017). However, the interspecific relationships within the genus remain unknown. Furthermore, Lycopodiaceae is ranked sixth among the most hybridization-prone plant families (Whitney et al., 2010) and there exists strong morphological evidence that *Dendrolycopodium* fits this trend. Hickey (1978) noted the existence of *Dendrolycopodium* plants that were intermediate for supposedly diagnostic traits, such as leaf ranking and angle of leaf divergence from the main stem (see Figure 1 for an example). This is consistent with ours and others’ field observations (Haines, 2003; Weston Testo, per. comm.). However, Hickey stated, “while it is impossible to prove the existence of hybrids in this species group, the secondary morphological evidence certainly suggests that hybridization does occur” (Hickey, 1978, p. 48).

Finding evidence of these hybrids and determining the species relationships is no longer such an impossible feat. With modern DNA sequencing technologies, we can now understand the population dynamics and evolutionary history of organisms, even those with notoriously cryptic reproduction, better than ever before. In this study, we employ double-digest restriction-site associated DNA sequencing (RADseq), together with a draft genome assembly, to begin to clarify the species relationships in the genus *Dendrolycopodium*. We demonstrate that these methods serve as a vital tool in studying the evolutionary history of Lycopodiales, despite their large genome sizes.

## Materials and Methods

### Collections and DNA Extractions

**FIGURE 2:**
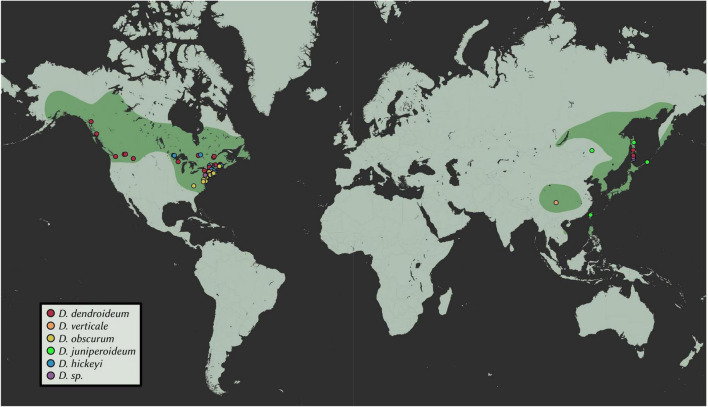
Geographic distribution of samples. Points represent samples used in this study; estimated ranges are highlighted. Map tiles by Stamen Design, under CC BY 3.0. Data by OpenStreetMap, under ODbL.

A total of 102 samples were included in this study. Fresh materials were collected from central New York, with a few branches from each plant silica-dried for DNA extractions. From these, extractions were conducted on 30 *Dendrolycopodium* individuals and 3 outgroup samples. To achieve the best geographic coverage, herbarium specimens and silica-dried materials collected after 1995 were also sampled (69 samples total, all *Dendrolycopodium*) (Figure 2 and Supplementary Table 1). Samples that did not clearly key to a species or showed intermediate morphology were called *Dendrolycopodium* sp. (D. sp.). Dried tissue samples (0.01–0.03 g) were added to a 2 mL tube with two, 3.5 mm stainless steel balls. Tubes were then submerged in liquid nitrogen and shaken at 1,500 strokes per minute on a MiniG 1600 (SPEX Sample Prep). DNA extractions were completed using a modified CTAB protocol (Saghai-Maroof et al., 1984). Extraction concentrations were quantified using the HS Qubit Kit (Invitrogen, MA, United States).

### Draft Genome Sequencing and Assembly

Illumina genomic library was prepared from *Dendrolycopodium obscurum* (sample name: Dob22; Supplementary Table 1) using TruSeq DNA PCR-free library kit, and sequenced on a NovaSeq 6000 using paired ends and a read length of 150 bp. Reads were screened for organelle and obvious contaminates removed. The filtered reads were then assembled with HipMer *De Novo* Assembler (v. 1.1-27-g69eb6141, Georganas et al., 2015) with parameters “–k 101,” which assembles paired Illumina data into non-redundant sequence contigs.

### Restriction-Site Associated DNA-Sequencing Library Preparation and Sequencing

A double-digest RADseq libraries were prepared based on a protocol by Parchman et al. (2012), with modifications by Nicolas Devos and Duncan Hauser. Samples were digested using EcoRI and MseI restriction enzymes, ligated to barcoded adaptors, and amplified (see Supplementary Notes for protocol details). The HS Qubit kit was used to quantify sample recovery after PCR. A total of 50 ng from each sample were then pooled and SparQ beads (QuantaBio, MA, United States) were used for a 250–500 bp size selection. The resultant library was submitted to Cornell’s Genomics Facility and sequenced on Illumina NextSeq 500 (150 bp single-end, high output flowcell).

### Restriction-Site Associated DNA-Sequencing Data Processing

Raw sequence files were processed using Cutadapt (v. 1.18, Martin, 2011) to remove poly-G tails resulting from 2-color Illumina chemistry, Illumina adapters, and poly-A tails as well as to demultiplex (allowing up to one mismatch in each barcode; commands: cutadapt –nextseq-trim = 20; cutadapt -a “A{100}” –minimum length 60; cutadapt -e 0.15 –no-indels -g file:barcodes). Trimmed, demultiplexed sequences were then processed using iPyrad (v. 0.7.30, Eaton, 2014). To map the cleaned reads, we used the *D. obscurum* draft genome (Table 1) as the reference. Reads were clustered at 88% identity with a minimum depth of 6 reads and a maximum depth of 10,000 reads within individual samples. For phylogenetic analyses, we kept loci that were present in at least four samples. Because STRUCTRUE is especially sensitive to missing data, we applied a more stringent filter, only including loci that were shared by at least 20 samples. Poorly performing samples (< 1,000 loci) were removed after processing in iPyrad.

**TABLE 1:** *Dendrolycopodium obscurum* draft genome statistics.

Estimated genome size	4.79 Gb
Assembled genome size	4.55 Gb
% Repeats	40%
Scaffold number	3,484,908
Scaffold N50	4.3kb
Contig number	4,321,281
Contig N50	2.8 kb
Scaffold contig coverage	97.97%
% gap	2

### STRUCTURE Analyses

STRUCTURE files were created by iPyrad and analyzed in STRUCTURE (v. 02.3.4, Pritchard et al., 2000). Five datasets were used for STRUCTURE analyses: one containing all *Dendrolycopodium* samples, and four datasets for hierarchical analyses (Janes et al., 2017) of each of the major clades detected in the initial STRUCTURE output. All STRUCTURE analyses were run for 100,000 Markov chain Monte Carlo (MCMC) generations (50,000 burn-in and 50,000 analysis generations), under default parameters with admixture for *K* = 2 to *K* = 5 with 3 replicates at each *K*-value. Outputs were viewed using STRUCTURE HARVESTER (Earl and vonHoldt, 2012). Optimal *K*-values were assessed by selecting the value for which the slope of the natural log probability was highest (Evanno et al., 2005). STRUCTURE plots were created using STRUCTURE PLOT (v. 2.0, Ramasamy et al., 2014).

### Phylogenetic Analyses

Maximum likelihood phylogenies were inferred for the unlinked SNPs data output (from iPyrad) using RAxML-HPC v.8.2.12 on XSEDE (Stamatakis, 2014) with a general time reversible model of nucleotide substitution drawing rates from the CAT approximation of rate heterogeneity (GTRCAT). Putative hybrid individuals (based on our STRUCTURE analysis) were removed prior to the phylogenetic inference. To search for the best tree, 50 independent runs with different starting points were executed. Branch supports were assessed with 500 non-parametric bootstrap replicates and by the Quartet Sampling approach (Pease et al., 2017). Tree files were processed in FigTree (Rambaut, 2012). Phylogenetic network was reconstructed by NeighborNet (Bryant and Moulton, 2004).

## Results

### Draft Genome and Restriction-Site Associated DNA-Sequencing Statistics

A total of 364.6 Gb cleaned and filtered Illumina reads were generated from the Dob22 sample of *Dendrolycopodium obscurum*. The HipMer assembly resulted in a draft genome that is 4.461 Gb in size with 3.513 Gb of contigs > 1 kb (contig N50 of 4.4 kb) (Table 1). RADseq yielded around 563 million reads from 102 samples. After cleaning and demultiplexing, around 470 million reads remained. A total of 13 poor performing samples were removed after processing in iPyrad, resulting in the following samples counts per species: 2 *Dendrolycopodium verticale*; 5 *D. juniperoideum*; 28 *D. obscurum*; 14 *D. hickeyi*; 24 *D. dendroideum*; 13 *D. sp.* samples that were not clearly identifiable; and 3 outgroup samples (*Lycopodium clavatum, Spinulum annotinum*, and *Diphasiastrum digitatum*).

Silica-dried samples had significantly more loci recovered compared to herbarium specimens (Supplementary Figure 1), which is consistent with the fact that DNA is more degraded in herbarium specimens.

### Population Structure

**FIGURE 3:**
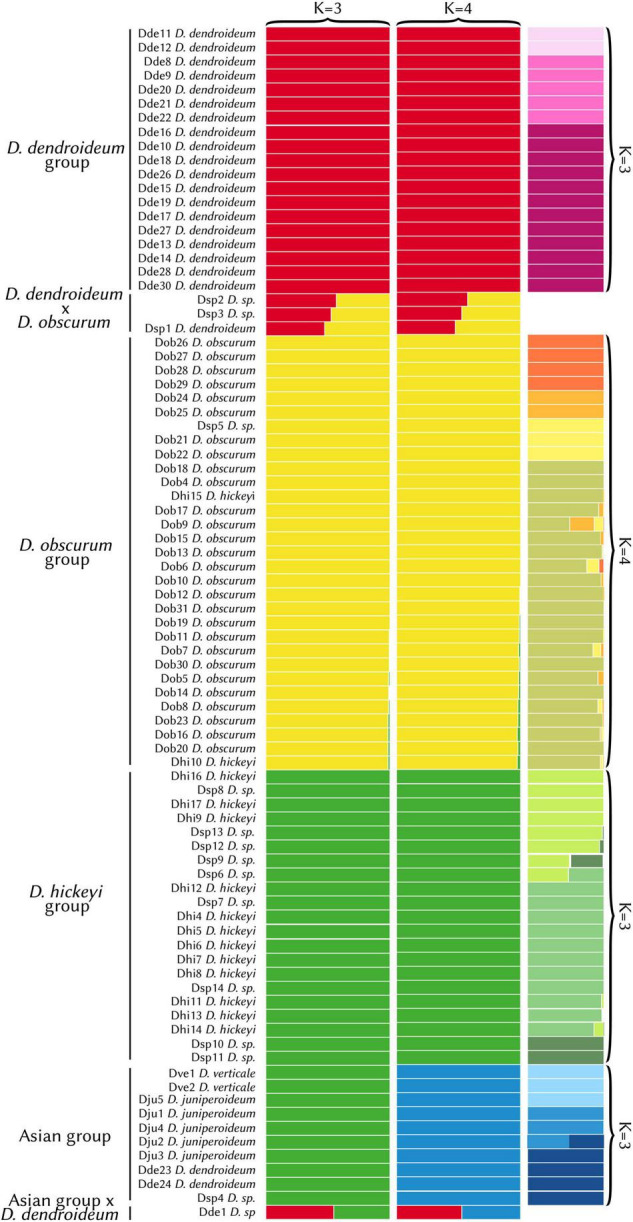
STRUCTURE results (*K* = 3 vs. *K* = 4). The hierarchical results are shown in the far-right column. Detailed sample information can be found in Supplementary Table 1. With *K* = 4, the Asian group (blue) was segregated, which is consistent with the phylogenetic results (see Figures 4, 5).

The STRUCTURE analysis had an optimum at *K* = 3. However, there was little difference in the likelihood values between *K* = 3 and *K* = 4 (Supplementary Table 2). The *K* = 3 result fails to separate the Asian samples as a distinct group, likely due to the limited sampling in the area. Because *K* = 4 distinguishes this clade, which is evident in the phylogeny (see below), it reflects a more accurate grouping (Figure 3). The first group consists entirely of *D. dendroideum* samples (“*D. dendroideum* group”). The second (“*D. obscurum* group”) includes mostly *D. obscurum*, except for two *D. hickeyi* samples and one *D.* sp. The third group (“*D. hickeyi* group”) includes 9 *D.* sp. samples and the remaining *D. hickeyi* samples. The fourth group encompassed all the Asian samples including *D. verticale, D. juniperoideum, D. dendroideum*, and *D.* sp. (the “Asian group”). Three hybrids were detected between the *D. dendroideum* and *D. obscurum* groups and one between the *D. dendroideum* and Asian groups.

Hierarchical STRUCTURE analyses for each of the four clades detected subdivisions. In the *D. dendroideum* group, three subgroups were detected, with no admixture detected between them. In the *D. obscurum* group, four subgroups were detected, with some admixture. In the *D. hickeyi* group, three subgroups were detected with some clearly admixed samples. In the Asian group, three subgroups were detected, with one sample a clear mix between the second and third subgroup (Figure 3).

### Phylogenetic Relationship

**FIGURE 4:**
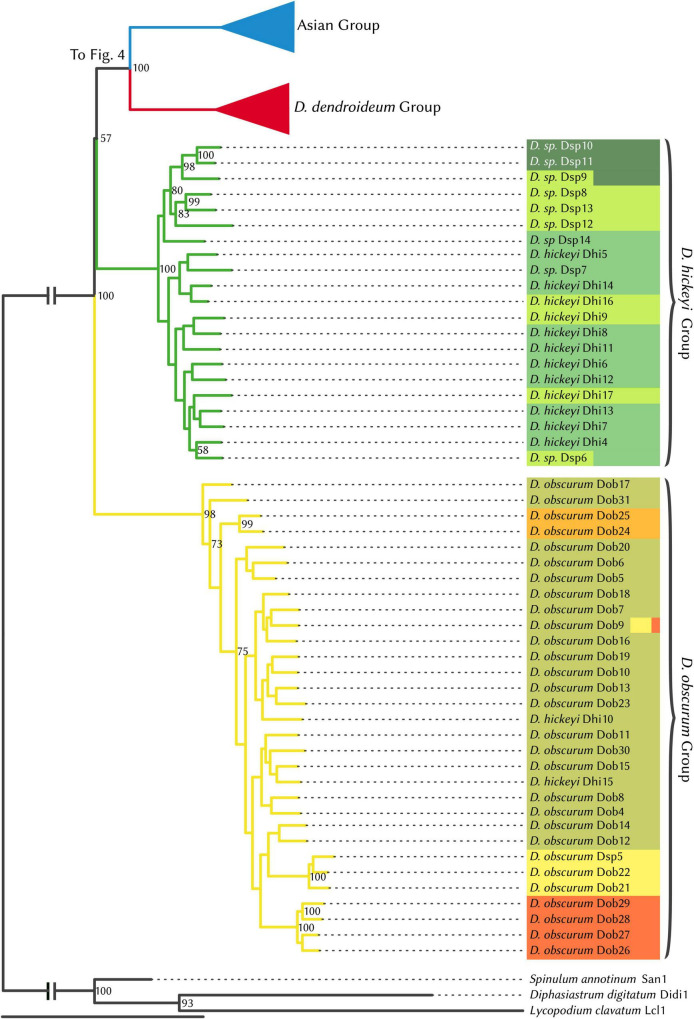
RAxML phylogenetic tree, *Dendrolycopodium hickeyi* group, and *D. obscurum* group. The best tree produced from 50 alternate starting runs (likelihood = –2703881.2); node labels represent bootstrap values (values < 50 not pictured). Colors correspond to STRUCTURE groupings (see Figure 3).

**FIGURE 5:**
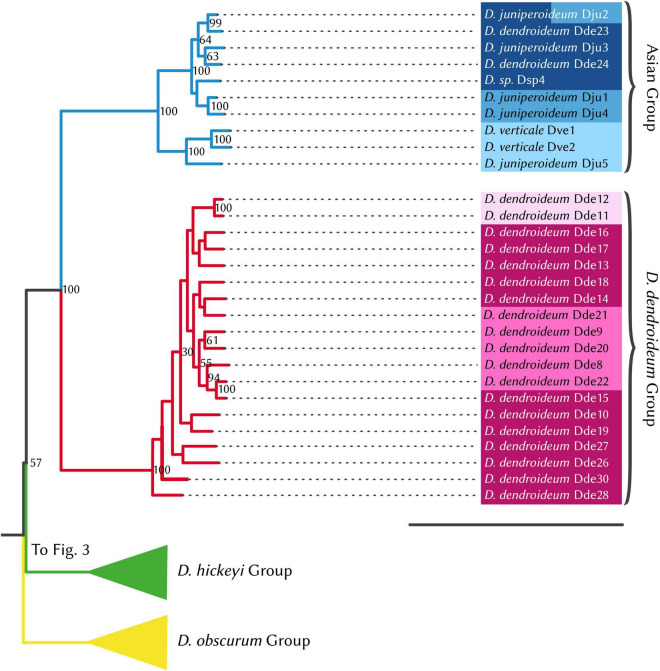
RAxML phylogenetic tree, *Dendrolycopodium dendroideum* group and Asian group. The best tree produced from 50 alternate starting runs (likelihood = –2703881.2); node labels represent bootstrap values (values < 50 not pictured). Colors correspond to STRUCTURE groupings (see Figure 3).

Prior to phylogenetic analyses, the four hybrid individuals identified by STRUCTURE analysis were excluded. The maximum likelihood phylogeny (Figures 4, 5) supports four major clades, which are reflected in the STRUCTURE data. The sister relationship between *D. dendroideum* and the Asian samples is well supported. Within the Asian samples, *D. juniperoideum*, however, is not recovered as monophyletic. The positions of the *D. hickeyi* group and *D. obscurum* group are unclear, with a low BS support of 57 and very short backbone branches. This uncertainty can also be visualized in the NeighborNet graph, which shows a web-like structure around the branches connecting to the *D. hickeyi* and *D. obscurum* groups (Supplementary Figure 2). Using the Quartet Sampling method, we also found a low quartet concordance score at the node after *D. obscurum* split (Supplementary Figure 2), suggesting the presence of conflicting phylogenetic signal.

## Discussion

### Species Delineation

Overall, both the phylogeny and the STRUCTURE results indicate that *D. dendroideum, D. obscurum*, and *D. hickeyi* represent distinct genetic entities. The relationship among these entities, however, could not be resolved. There are apparently conflicting phylogenetic signals regarding to the placements of the *D. hickeyi* group and *D. obscurum* (Figure 4 and Supplementary Figure 2).

As it currently stands, the morphotaxonomy is a somewhat reasonable reflection of species boundaries, however, it is insufficient on its own. Both Dhi 10 and Dhi15 key very clearly to *D. hickeyi*, yet they fall into the *D. obscurum* clade (Figures 3, 4). Additionally, many samples in the *D. hickeyi* clade cannot clearly be identified as such from the current species descriptions and keys; some even show an intermingling of traits specific to other species despite not being recovered as hybrids.

Phylogenetic and STRUCTURE results also indicate that all the Asian samples included in this study represent a separate distinct genetic entity comprised of a few described species: *D. verticale, D. juniperoideum*, and *D. dendroideum*. Despite being clearly morphologically different from the other described taxa, *D. juniperoideum* is not recovered as monophyletic (Figure 5). Further sampling and a thorough review of morphology should be conducted to evaluate the validity of *D. juniperoideum*. Additionally, samples identified as *D. dendroideum* in this clade appear to be a separate genetic entity from those in the main *D. dendroideum* group. Again, more sampling and a detailed morphological review of the *D. dendroideum* in Asia is required to determine the taxonomic fate of these plants.

### Hybrids

Four interspecific hybrids were identified from STRUCTURE analyses. All *D. dendroideum x obscurum* samples (Dsp1, Dsp2, and Dsp3) have a chimeric appearance, with some parts of the plant matching the characters of *D. dendroideum* and others matching *D. obscurum* (see Figure 1). Dde1 (*D. denroideum* × Asia group), however, simply resembles *D. dendroideum*. From this data, it is impossible to determine if these hybrids are of homoploid or polyploid origin. It has been suggested that hybrids in two Lycopodiaceae subfamilies, Huperzioideae and Lycopodielloideae, tend to be polyploid, but are predominantly homoploids in the remaining subfamily that includes *Dendrolycopodium*, Lycopodioideae (Wagner et al., 1985; Wagner, 1992). Specifically, homoploid hybrid speciation has been most explored and supported in *Diphasiastrum* (also in subfamily Lycopodioideae) (Wilce, 1965; Holub, 1975; Wagner and Beitel, 1993; Stoor et al., 1996; Aagaard et al., 2009). Phylogenetically based proclivities for homoploidy vs. polyploidy could provide insight into the reproductive mechanisms and genome structural elements that shape hybridization, speciation, and broad evolutionary trajectory in lycophytes.

### Biogeography

**FIGURE 6:**
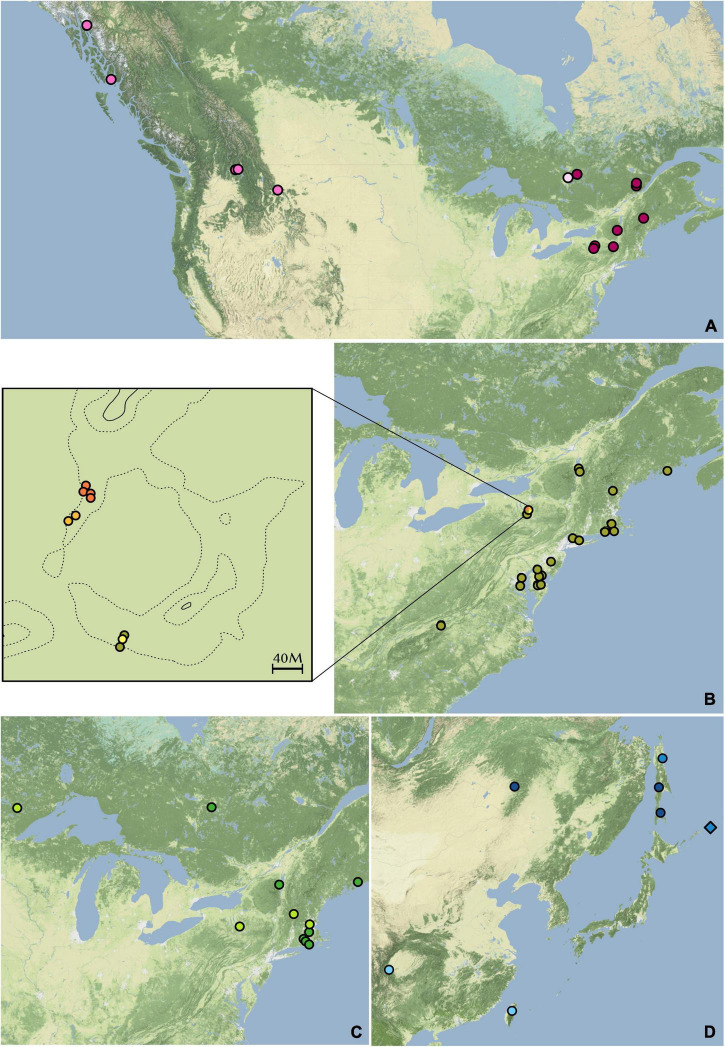
Distributions of hierarchical STRUCTURE subgroups. (A) *Dendrolycopodium dendroideum* group. (B) *D. obscurum* group. The enlarged area is McLean Bogs, New York. (C) *D. hickeyi* group. (D) The Asian group. Colors correspond to the hierarchical STRUCTURE results in Figure 3.

Many of the finer groupings resulting from the hierarchical STRUCTURE analysis are reflected in their distribution. Within the *D. dendroideum* group, Dde8, Dde9, Dde20, Dde21, and Dde22 are all from western North America (Figure 6A). On the other hand, Dde15 is also from western North America but is more genetically similar to the remainder of the samples from eastern North America.

Within the *D. obscurum* group, all four genetic subgroups discovered in the hierarchical STRUCTURE analysis occur in McLean Bogs, New York (42.54795, –76.26633), with three of the four subgroups only being found there (Figure 6B). It is possible that this area acted as a refugium during glaciation, thus more ancient genetic diversity was preserved here. A detailed sampling of *D. obscurum*, with more evenly distributed coverage of its range, would be required to determine if these genetic subgroups occur elsewhere or if there are more pockets of hidden genetic diversity.

Within the *D. hickeyi* group, there does not appear to be a relationship between geographic distribution and the genetic subgroups described by the hierarchical STRUCTURE analysis (Figure 6C). All the subgroups occur in central New York, where around nearly half of the *D. hickeyi* group samples were collected.

Finally, the samples within the Asian group split into two distinct geographic subgroupings with Dve1, Dve2, and Dju5 in the more southern subgroup and Dju1, Dju2, Dju3, Dde23, Dde24, and Dsp4 located further north (Figure 6D). As stated previously, *D. juniperoideum* does not appear to be monophyletic and is represented in both of these subgroups. Once again, a thorough sampling throughout the range of *Dendrolycopodium* in Asia is crucial to elucidating the genetic diversity of this region and reevaluating the taxonomy.

### Taxonomic Recommendations

The North American *D. obscurum, D. dendroideum*, and *D. hickeyi* are supported as monophyletic by the genetic data and thus should remain valid taxa. Morphometric analyses should be revisited to determine if there exist any consistent characters that can be used to reliably differentiate them and their hybrids. However, the taxonomy of the Asian *Dendrolycopodium* must be revised because *D. juniperoideum* is not supported as monophyletic and the Asian *D. dendroideum* does not clade with the bulk of *D. dendroideum*. As such, we tentatively recommend that all members of this clade be renamed *D. juniperoideum* as it predates the name *D. verticale*. We recognize that this creates a group that cannot be defined with even the clearest of morphological characters (e.g., the number of leaves per rank differs between *D. juniperoideum and D. verticale*) and must instead rely on geography. We suspect that further sampling may warrant the distinction of species between the northern (i.e., Russia, Japan, Korea) and southern (i.e., China, Taiwan) extent of the range, in which case, the name *D. verticale* should be resurrected for the southern group. However, the data presented here lack the sampling, as well as morphological features, to support this.

## Data Availability Statement

The datasets presented in this study can be found in online repositories. The names of the repository/repositories and accession number(s) can be found below: https://www.ncbi.nlm.nih.gov/, PRJNA821109; https://www.ncbi.nlm.nih.gov/, PRJNA822420. The draft genome assembly can be found at https://doi.org/10.6084/m9.figshare.19448483.v1.

## Author Contributions

AP, DH, and F-WL planned the study. AP collected fresh material and sampled herbarium specimens, completed DNA extractions and RADseq library preparation. AP and DH conducted data processing and analysis. MK, JS, and JG sequenced and assembled the *Dendrolycopodium obscurum* draft genome. AP wrote the manuscript with input from DH and F-WL. All authors contributed to the article and approved the submitted version.

## Conflict of Interest

The authors declare that the research was conducted in the absence of any commercial or financial relationships that could be construed as a potential conflict of interest.

## Publisher’s Note

All claims expressed in this article are solely those of the authors and do not necessarily represent those of their affiliated organizations, or those of the publisher, the editors and the reviewers. Any product that may be evaluated in this article, or claim that may be made by its manufacturer, is not guaranteed or endorsed by the publisher.
