# Supplementary material for: Re-evaluating the Systematics of Dendrolycopodium Using Restriction-Site Associated DNA-Sequencing
Source: Front Plant Sci. 2022 Jun 9;13:912080. doi: 10.3389/fpls.2022.912080 (PMC9218423; doi:10.3389/fpls.2022.912080)
Supplement: Supplementary file 1 [file Table_1.DOCX]

**Supplementary Notes: ddRAD-seq protocol**

*Digestion:* For each sample, the following reagents were mixed in a PCR tube on ice: 10 µL DNA (or 500ng, whichever volume was greater); 1µL EcoRI (20,000 units/mL); 2µL MseI (10,000 units/mL); 5µL 10x CutSmart buffer (New England Biolabs); PCR water to a total volume of 50µL. Samples were incubated at 37°C for three hours, followed by 65°C for 10 minutes.

*MseI oligo preparation:* MseI oligos (Appendix 9) had to be annealed before ligation by combining 10µL MseI1 oligo (100µM stock), 10µL MseI2 oligo (100µM stock), and 80µL PCR water in a PCR tube. The tube was incubated with the following steps: (1) 95°C for 5 minutes; (2) 80°C for 30 seconds, looped 58 times with temperature reducing by 1°C each loop; (3) 22°C for 1 minute; (4) 95°C for 5 minutes; and (5) 80°C for 30 seconds, looped 58 times with the temperature reducing by 1°C each loop.

*Ligation*: For each sample, the following reagents were added to a PCR tube on ice: 2µL 10X T4 ligase buffer (New England Biolabs); 1µL prepared MseI adapter (10µM); 0.4µL T4 ligase (New England Biolabs); 3.6µL PCR water; 12µL digested DNA; and 1µL barcoded EcoRI adapter. Tubes were incubated at 23°C for 1 hour.

*PCR*: For each sample, the following reagents were added to a PCR tube on ice: 7.65 µl PCR water; 4µL 5x Phusion HF buffer (New England Biolabs); 4µL mixed dNTPs (1mM each); 2µL 2.5µM pooled IllPCR primers; 0.15µL DMSO; 0.2µL Phusion DNA polymerase (New England Biolabs); 2µL digested, ligated DNA product. Tubes were incubated in the following steps: (1) 98°C for 30 seconds; (2) 98°C for 20 seconds; (3) 60°C for 30 seconds; (4) 72°C for 40 seconds; loop steps (2), (3), and (4) 29 times; and (5) 72°C for 10 minutes.

**ddRAD-seq primer and oligo sequences**

*MseI1 oligo:* 5’ GCAGAAGACGGCATACGAGCTCTTCCCATCTG 3’

*MseI2 oligo:* 5’ TACAGATCGGAAGAGCTCGTATGCCGTCTTCTGCTTG 3’

*IllPCR1 oligo:* 5’ A*A*TGATACGGCGACCACCGAGATCTACACTCTTTCCCT

ACACGACGCTCTTCCGATCT 3’

*IllPCR2 oligo:* 5’ C*A*AGCAGAAGACGGCATACGAGCTCTTCCGATCTGT

AAG 3’

*=phosphothiolated base
