# Supplementary figures and images for: Re-evaluating the Systematics of Dendrolycopodium Using Restriction-Site Associated DNA-Sequencing
Source: Front Plant Sci. 2022 Jun 9;13:912080. doi: 10.3389/fpls.2022.912080 (PMC9218423; doi:10.3389/fpls.2022.912080)

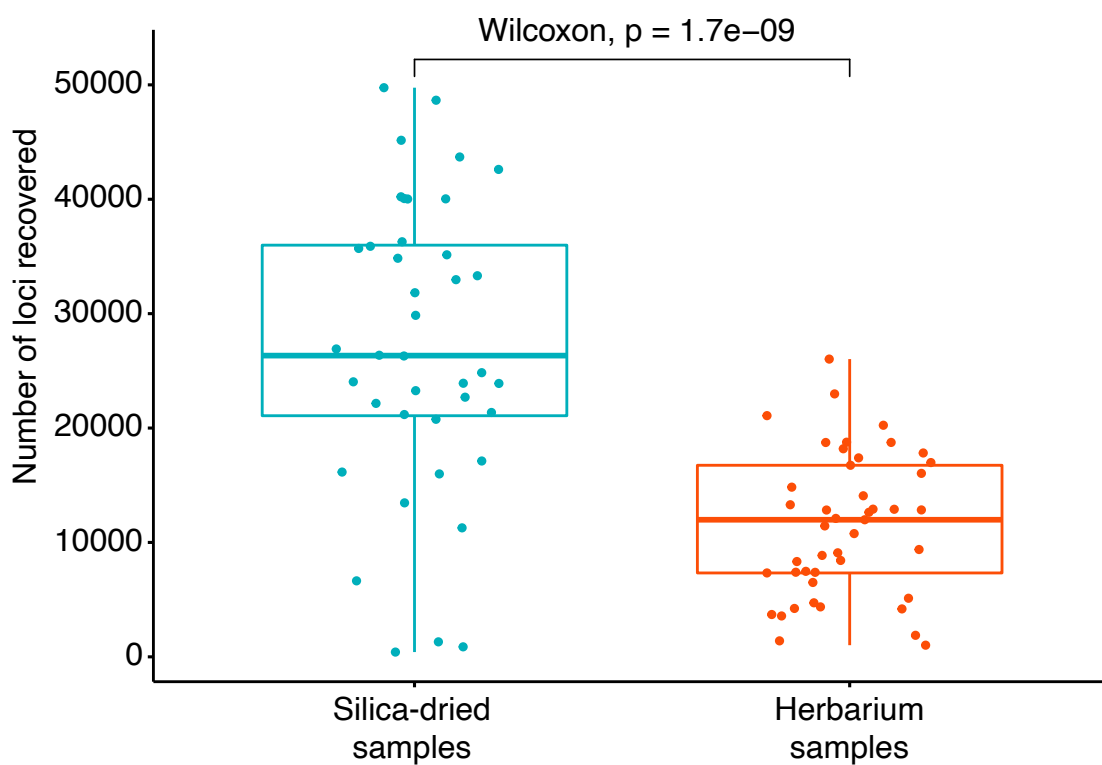

Supplementary Figure S1. Comparison of loci recovery from silica-dried and herbarium samples

Supplement: Supplementary file 4 [file Data_Sheet_1.PDF]
