# Supplementary material for: Re-evaluating the Systematics of Dendrolycopodium Using Restriction-Site Associated DNA-Sequencing
Source: Front Plant Sci. 2022 Jun 9;13:912080. doi: 10.3389/fpls.2022.912080 (PMC9218423; doi:10.3389/fpls.2022.912080)
Supplement: Supplementary file 5 [file Data_Sheet_2.PDF]

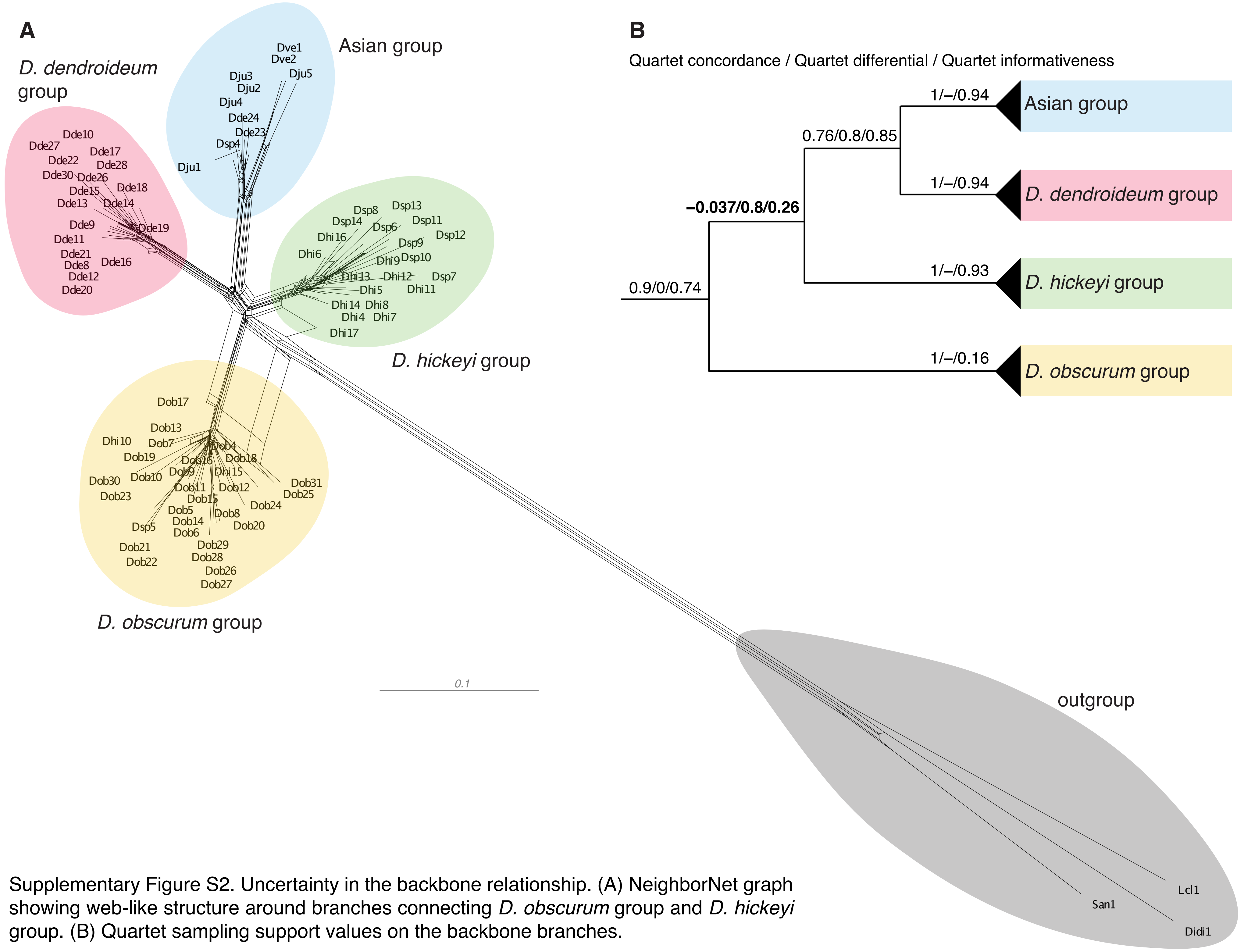


Supplementary Figure S2. Uncertainty in the backbone relationship. (A) NeighborNet graph showing web-like structure around branches connecting *D. obscurum* group and *D. hickeyi* group. (B) Quartet sampling support values on the backbone branches.
